# Heterogeneity in clinically diagnosed type 1 diabetes: characterising a unique cohort with maintained C-peptide secretion in Ghana

**DOI:** 10.1007/s00125-025-06576-3

**Published:** 2025-11-01

**Authors:** Wilfred Aniagyei, Osei Sarfo-Kantanka, Sumaya Mohayideen, Monika M. Vivekanandan, Ernest Adankwah, Shadrack O. Asibey, Agnes O. Boateng, Elisabeth Owusu, Joseph F. Arthur, Augustine Yeboah, Hubert S. Ahor, Dorcas O. Owusu, Maximilian Huttasch, Yanislava Karusheva, Volker Burkart, Robert Wagner, Michael Roden, Vera Balz, Jürgen Enczmann, Maike Sterzenbach, Sebastian Kummer, Thomas Meissner, Diran Herebian, Ertan Mayatepek, Marc Jacobsen, Richard O. Phillips, Julia Seyfarth

**Affiliations:** 1 Division of General Pediatrics, Neonatology and Pediatric Cardiology, Medical Faculty and University Hospital Duesseldorf, Heinrich Heine University Duesseldorf, Duesseldorf, Germany; 2 Kumasi Centre for Collaborative Research in Tropical Medicine (KCCR), Kumasi, Ghana; 3 Komfo Anokye Teaching Hospital, Kumasi, Ghana; 4 School of Medicine and Dentistry, College of Health Sciences, Kwame Nkrumah University of Science and Technology (KNUST), Kumasi, Ghana; 5 Institute for Clinical Diabetology, German Diabetes Center, Leibniz Center for Diabetes Research at Heinrich Heine University Düsseldorf, Düsseldorf, Germany; 6 Department of Endocrinology and Diabetology, Medical Faculty and University Hospital Düsseldorf, Heinrich Heine University Düsseldorf, Düsseldorf, Germany; 7 German Center for Diabetes Research, Partner Düsseldorf, München-Neuherberg, Germany; 8 Institute for Transplantation Diagnostics and Cell Therapeutics, Medical Faculty, University Hospital, Duesseldorf, Germany

**Keywords:** Autoantibodies, Autoimmunity, HLA, Ketosis-prone type 2 diabetes, Serum biomarker, Type 1 diabetes

## Abstract

**Aims/hypothesis:**

In sub-Saharan Africa, type 1 diabetes is typically diagnosed clinically, which can be challenging due to atypical diabetes presentations such as ketosis-prone type 2 diabetes or type 2 diabetes in the absence of overweight and obesity. C-peptide, a marker of residual insulin secretion capacity, is crucial for understanding these variations but understudied in the region. Here, we investigated whether C-peptide measurement and concomitant genetic, autoimmune and metabolic characterisation of individuals with clinically diagnosed type 1 diabetes confirm diabetes classification and highlight population-specific features.

**Methods:**

In this case–control study from Ghana, we recruited 266 individuals with clinically diagnosed and insulin-treated long-term type 1 diabetes and 266 healthy control individuals. We compared clinical features, HLA class II haplotypes, autoantibodies, and inflammatory and metabolic serum profiles across control and patient groups classified by random C-peptide levels: low (<0.2 nmol/l), mid (0.2–0.6 nmol/l) and high (>0.6 nmol/l).

**Results:**

Only 28.9% of individuals with clinically diagnosed type 1 diabetes had low C-peptide concentrations. They were the youngest and leanest group, with higher frequencies of HLA class II risk haplotypes and GAD and ZnT8 autoantibodies compared with all other groups. By contrast, 34.6% and 36.5% had mid-range or high C-peptide levels, respectively. These subgroups resembled the control group in terms of low autoantibody titres and one protective HLA class II haplotype. Ketosis at onset was most prevalent in individuals with high C-peptide. Serum proinflammatory biomarkers differed between individuals with diabetes and control participants, but not between C-peptide subgroups. Aromatic and branched-chain amino acids varied between diabetes subgroups and positively correlated with C-peptide levels.

**Conclusions/interpretation:**

Maintained C-peptide levels in two-thirds of individuals with long-term type 1 diabetes in Ghana, combined with the absence of autoantibodies and HLA risk association, highlight the necessity for better differentiation from atypical diabetes presentations to optimise patient care and improve health outcomes in resource-limited settings.

**Graphical Abstract:**

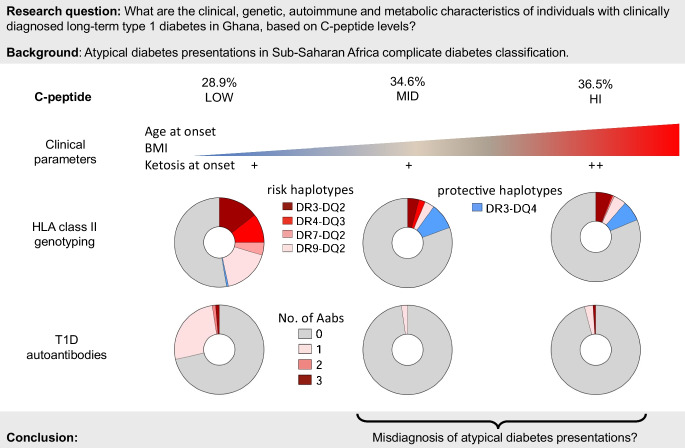

**Supplementary Information:**

The online version of this article (10.1007/s00125-025-06576-3) contains peer-reviewed but unedited supplementary material.


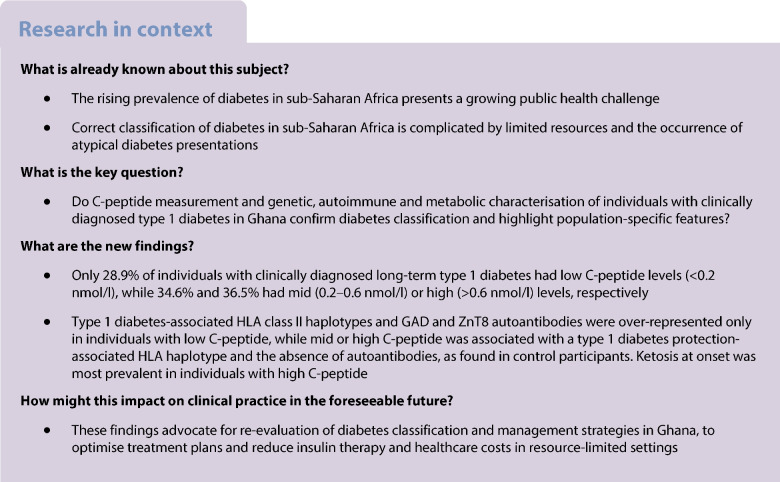


## Introduction

Diagnosis of type 1 diabetes in sub-Saharan Africa presents unique challenges distinct from those in Western countries. First, the clinical presentation of type 1 diabetes in Africa differs from that in other populations. Previous studies have suggested that the peak age of onset is later in sub-Saharan Africa, ranging from late adolescence to young adulthood (15–30 years) compared with 10–15 years in Europeans [1, 2]. Moreover, individuals with type 1 diabetes often present with advanced complications at onset, which may be due to delays in diagnosis and limited access to healthcare resources [3]. Diagnosis of type 1 diabetes in sub-Saharan Africa is further complicated by atypical diabetes variants, including ketosis-prone type 2 diabetes (KPD), malnutrition-related diabetes and type 2 diabetes in non-overweight individuals [1, 4–6]. These diabetes entities specifically occurring in Africa and people of African ancestry have received limited research. Clinical features overlapping with those of classic type 1 diabetes complicate diagnosis, especially in KPD, which initially presents as severe ketosis or ketoacidosis and insulin dependency, which potentially misdiagnoses individuals as having type 1 diabetes.

In resource-limited settings lacking comprehensive diagnostic tools, the classification of diabetes is typically based on clinical symptoms [1]. Even when available, autoantibodies may only partially support diagnosis in sub-Saharan Africa, as studies have consistently described lower seropositivity compared with that in Western countries [1]. Moreover, population-specific autoantibody cutoffs necessary for interpretation are largely unavailable [7]. However, whether lower levels of antibodies are due to misclassification or a true difference in phenotype of autoimmune diabetes in Africa is unclear. C-peptide, a marker of residual insulin secretion, could confirm insulin deficiency in type 1 diabetes but is rarely tested in sub-Saharan Africa. It has proved to be useful in determining the cause of diabetes [8] and its measurement leads to relevant reclassifications in clinically diagnosed long-term type 1 diabetes, even in high-resource settings [9].

Comprehensive studies on clinically diagnosed type 1 diabetes in sub-Saharan Africa are limited. This includes Ghana, where limited data exist on the disease’s prevalence and apparent later age of onset [2, 10]. It is uncertain whether the local clinical diagnosis reflects the typical disease pattern in Europeans, with established diagnostic parameters such as absolute insulin deficiency, autoantibodies and genetic HLA class II background [11]. This study investigated whether measuring random C-peptide levels and characterising autoimmune factors (HLA genetics and type 1 diabetes autoantibodies) and non-autoimmune factors (inflammatory serum proteins and the amino acid profile) in individuals with clinically diagnosed, long-term type 1 diabetes in Ghana confirms the diabetes classification and highlights population-specific characteristics. Besides established autoimmune factors, the investigation of non-autoimmune pathways such as those involving inflammatory serum proteins and amino acids could help further characterise potentially misclassified people with diabetes, since both have been shown to differ between diabetes types and are associated with insulin resistance [12–16]. Thus, we aimed to enhance our understanding of regional type 1 diabetes presentation and address misdiagnosis due to atypical variants, ultimately aiming to improve patient care in resource-constrained settings.

## Methods

### Study population

We recruited 266 individuals with clinically diagnosed, long-term type 1 diabetes (disease duration >1 year) and 266 healthy control individuals from August 2021 to February 2023 at the Diabetes Clinic of the Komfo Anokye Teaching Hospital (KATH), the main referral clinic for diabetes in the middle belt of Ghana. Diagnosis of diabetes followed the ADA criteria [17] and classification as type 1 diabetes was based on clinical symptoms suggestive of insulin deficiency such as polyuria, polydipsia, weight loss, ketonaemia/ketonuria or marked hyperglycaemia nonresponsive to oral glucose-lowering drugs. Confirmatory testing at onset of disease using type 1 diabetes autoantibody screening and C-peptide measurement was only performed in ambiguous cases. All participants were on persistent insulin therapy. The control group comprised healthy individuals without a diabetes diagnosis who had a negative history of autoimmune or systemic inflammatory diseases and were age- and sex-matched (age ±6 years) to long-term diabetes individuals. Relatives of people living with diabetes were excluded from the control group. Demographics of study participants are given in Table 1. In addition, individuals with new-onset diabetes (*n*=54, disease duration <1 year) were recruited (characteristics in ESM Table 1).

**Table 1:** Demographics of study participants

Characteristic	Control	T1D	*p* value
Number, *n*	266	266	
Female/male (% female)	182/84 (68)	182/84 (68)	>0.99
Age, years	33 (18–51)	36.5 (20–52)	0.338

Data are presented as median (IQR) unless otherwise specified

Categorical variables were compared using χ^2^ test, and continuous variables using one-way ANOVA, as appropriate. Statistical significance was defined as* p*<0.05

Written informed consent was obtained from all study participants (older than 14 years) and their legal guardians. The study received approval from the Ethics Committee Board (KATH IRB/AP/081/20) at KATH in Kumasi, Ghana, and has been carried out in accordance with the Declaration of Helsinki.

### Clinical and biochemical data

Medical history was documented in questionnaires. Anthropometric measurements were recorded. For adults, BMI classification was calculated with categories defined as underweight (<18.5 kg/m^2^), normal weight (18.5 to <25 kg/m^2^), overweight (25 to <30 kg/m^2^) and obese (≥30 kg/m^2^). Children were classified according to their BMI percentiles defined by standardised growth charts (underweight <5.0 percentile, overweight 85.0-95.0 percentile, obesity ≥95.0 percentile) [18]. Non-fasting whole blood and serum samples were collected from each participant and stored at −80°C. HbA_1c_ was measured using HPLC. C-peptide serum measurements were performed by electrochemiluminescence immunoassay using the e801 module of a Cobas 8000 system (Roche Diagnostics Deutschland, Mannheim; detection limit <0.0066 nmol/l).

Random and non-fasting C-peptide was measured for practical reasons and since it is less influenced by confounders and more effective for distinguishing type 1 from type 2 diabetes [19]. The following cutoff values for C-peptide were used for stratification as recommended by the literature: LOW (<0.2 nmol/l) for absolute insulin deficiency, MID (>0.2 to <0.6 nmol/l) and HI for preserved insulin production capacity (>0.6 nmol/l) [8, 17, 20]. The demographics and clinical characteristics of the diabetes cohort, stratified by C-peptide, are presented in Table 2.

**Table 2:** Demographics and clinical characteristics of the diabetes subgroups

Characteristic	Diabetes subgroups			*p* value
	HI	MID	LOW	
Number, *n*	97	92	77	
Female/male (% female)	73/24 (75)	68/24 (74)	41/36 (53)	0.004
Age, years	46 (30–57)	39 (21–53)	23 (15–34)	<0.001
Onset age, years	37 (20–48)	26 (16–37)	14 (11–23)	<0.001
BMI classification, *n* (%)				
Underweight	5 (5)	10 (11)	23 (30)	
Normal	30 (31)	39 (42)	43 (56)	
Overweight	35 (36)	27 (29)	5 (6)	
Obese	27 (28)	16 (17)	6 (8)	
BMI, kg/m^2^	26.7 (23.5–30.8)	24.6 (21.56–27.9)	20.2 (18.2–22.8)	<0.001
HbA_1c_, mmol/mol	71.3 (54.8–96.6)	87.2 (65.1–109)	84.3 (65.1–101.9)	
HbA_1c_, %	8.6 (7.2–10.9)	10.2 (8.2–12.3)	9.9 (8.1–11.5)	0.005
Insulin dosage/kg, U/kg per day	0.6 (0.4–0.8)	0.6 (0.4–0.9)	0.8 (0.6–1.2)	0.001
Duration of disease, years	7 (3–13)	9 (4–14)	8 (5–12)	0.267

Data are presented as median (IQR) unless otherwise specified

BMI classification is shown as number of cases (%)

Categorical variables were compared using χ^2^ test, and continuous variables using Kruskal–Wallis and one-way ANOVA, as appropriate. Statistical significance was defined as* p*<0.05

### HLA typing

Genomic DNA was extracted from heparinised blood samples using the DNAQiamp 96 DNA Blood kit (Qiagen, Hilden, Germany) according to the manufacturer’s instructions. An amplicon-based approach using Illumina next-generation sequencing technology was used for genotyping HLA-DRB1, -DQA1 and -DQB1 as previously described [21].

### Autoantibodies

Serum samples were thawed from −80°C and autoantibodies relevant for type 1 diabetes were measured at the German Diabetes Center (DDZ, Düsseldorf, Germany). GAD antibodies were measured by a radioligand assay (cutoff 2 U/ml) [22], IA-2 by RIA (cutoff 2 U/ml) and ZnT8 by ELISA (cutoff ≥15 U/ml) (Medipan, Dahlewitz, Germany).

### Proteomic profiling

Serum samples were analysed using the Olink Proteomics Inflammation Panel, which is a multiplex proximity extension assay designed to quantify 92 key inflammatory proteins. The assay provides relative quantification of protein concentrations as normalised protein expression values. Quality control measures were implemented per the manufacturer’s instructions. Four samples did not pass quality control and were removed from the analysis. Proteins with more than 75% of values below the lower limit of detection were excluded from further analysis (*n*=6).

### Metabolites

Protein precipitation was performed by adding sulfosalicylic acid including isotopically labelled internal standards to the serum samples. After centrifugation, the supernatant was diluted with water (MS grade) and transferred for analysis. The targeted compounds tyrosine, phenylalanine, tryptophan, valine, leucine and isoleucine were analysed by ultra-performance liquid chromatography (UPLC)-MS/MS. The system consists of a UPLC I-Class (Waters) coupled to a tandem mass spectrometer Xevo-TQ-XS (Waters). Electrospray ionisation was performed in the positive ionisation. Chromatographic separation was performed using an amide column and mass spectrometric quantification of the compounds was carried out in the multiple reaction monitoring mode [23]. MassLynx software (v4.2; Waters, UK) was used for instrument control and data acquisition. Quantification analysis was performed using TagetLynx XS software (Waters, UK).

### Calculations and statistics

Statistical analyses and figures were conducted using GraphPad Prism v10 software (GraphPad Software, La Jolla, CA, USA) and RStudio version 4.3.0 software (RStudio, Boston, MA, USA; packages: Bridging Immunogenomic Data-Analysis Workflow Gaps (BIGDAWG)/OlinkAnalyse/ComplexHeatmap). Non-parametric tests were employed as the data did not follow normal distribution (confirmed by Shapiro-Wilk and Kolmogorov-Smirnov tests). The χ^2^ test of independence assessed the association between the categorical variables across all groups. Following a significant overall χ^2^ result, post hoc pairwise χ^2^ tests between every pair of groups were performed. The Mann–Whitney U (2 groups) and the Kruskal-Wallis tests (>2 groups) were utilised for continuous variables, applying Dunn’s correction for multiple comparisons. HLA-DRB1, -DQA1 and -DQB1 alleles and haplotypes were analysed using the BIGDAWG package (version 3.0.8), which calculates ORs and CIs using standard contingency table methods and χ^2^ tests, assuming independent observations. Calculations of the Hardy-Weinberg equilibrium, CIs, ORs and *p* values were performed. Allele and haplotype counts <5 in the study population were collapsed into the ‘binned’ category and excluded from the analysis. Overall differences in proteomic biomarkers of inflammation between all study groups were analysed with the Kruskal-Wallis test. The *p* values were adjusted for multiple comparisons using the Benjamini‒Hochberg method. Correlations were estimated using Spearman rank correlation coefficients and corresponding *p* values. Statistical significance was defined as a *p* value below 0.05.

## Results

### High residual insulin secretion capacity in the presumed type 1 diabetes cohort

**Fig. 1:**
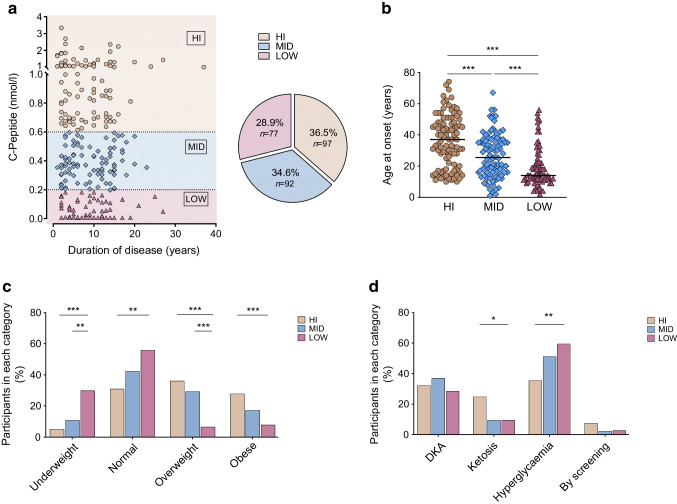
Clinical characteristics of the diabetes subgroups. (**a**) Distribution of individuals with diabetes into subgroups using C-peptide values (cutoffs 0.2 and 0.6 nmol/l) plotted against the duration of diabetes. Proportions and absolute numbers in diabetes subgroups (C-peptide HI, MID and LOW) are depicted as a pie chart. (**b**) Age at diabetes onset in diabetes subgroups is depicted. (**c**, **d**) Bar graphs are shown for (**c**) BMI classification into underweight, normal, overweight and obese participants and (**d**) presentation at diabetes onset (DKA, ketosis, hyperglycaemia, diagnosis by screening). Symbol plots are depicted with a median line. Differentiation by colour and shape plots is indicated: HI (yellow circles), MID (blue diamonds) and LOW (red triangles). The χ^2^ test was applied to categorical parameters, and the Kruskal–Wallis test was used for continuous variables; *p* values below 0.05 were considered significant. *p<0.05; **p<0.01; ***p<0.001

A total of 266 clinically diagnosed individuals with long-term type 1 diabetes (median disease duration of 8 years) were analysed for their random C-peptide as a surrogate parameter for residual beta cell function. Surprisingly, C-peptide values were very heterogeneous, and a considerable number of individuals had unexpectedly high levels (up to 3.34 nmol/l) (Fig. 1a). Only 28.9% (*n*=77) of participants had levels below the common cutoff of 0.2 nmol/l typical for long-term type 1 diabetes (hereafter referred to as C-peptide LOW). C-peptide levels between 0.2 and 0.6 nmol/l (termed C-peptide MID), which can occur in type 1 diabetes early after onset of the disease, but is not common after a disease duration of more than 3 years [8], were found in 34.6% (*n*=92). High C-peptide values above 0.6 nmol/l (termed C-peptide HI) typically seen in type 2 diabetes and indicative of a lack of insulin requirement [20] were detected in 36.5% (*n*=97). There was no correlation between C-peptide and the individual duration of disease (*p*=0.466, Fig. 1a).

### Clinical characterisation

Against the background of C-peptide heterogeneity, we compared the clinical characteristics of individuals with presumed type 1 diabetes classified by their C-peptide (Table 2). Only C-peptide LOW individuals showed the classical type 1 diabetes characteristics with an early onset of disease (median age at onset 14 years, Fig. 1b) and a low prevalence of overweight and obesity (6% and 8%, respectively, Fig. 1c). Individuals in the C-peptide MID and HI group were older at diabetes diagnosis compared with the C-peptide LOW group (median age at onset of 26 years and 37 years; *p*<0.001 and *p*<0.001, respectively, Fig. 1b) and in particular the C-peptide HI group was more likely to be overweight (36%) or obese (28%) than the C-peptide LOW group (*p*<0.001, respectively, Fig. 1c). HbA_1c_ values as indicators for the quality of blood glucose control were lower in the C-peptide HI group compared with the MID and LOW groups (Table 2). We also compared the clinical symptoms reported at disease onset. While diabetic ketoacidosis (DKA) at onset was similarly common in the three groups, ketosis at disease onset was highest in the C-peptide HI group (25%, *p*=0.01, Fig. 1d). These data showed differences in the clinical presentation of type 1 diabetes individuals, with the C-peptide LOW group most resembling classic type 1 diabetes, although ketosis at onset was most prominent in the C-peptide HI group.

### HLA class II genetic background

**Fig. 2:**
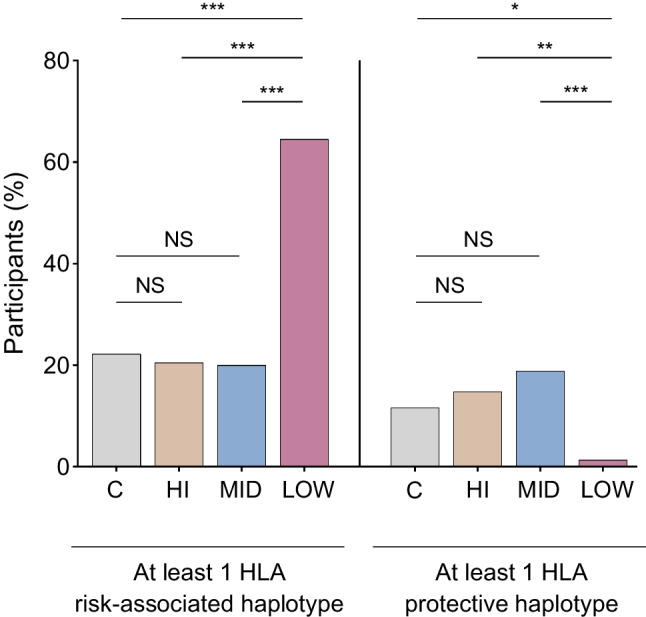
HLA haplotype distribution. The percentages of individuals carrying at least one risk-associated or protective HLA class II haplotype in the diabetes subgroups and control group (C) are represented by bar graphs. Significant differences in HLA haplotypes were calculated using the χ^2^ test. Only comparisons with significant *p* values (below 0.05) are depicted. *p<0.05; **p<0.01; ***p<0.001

To unravel the genetic HLA class II background of presumed type 1 diabetes in Ghana and to assess C-peptide-associated HLA differences, we genotyped individuals with diabetes and control individuals at the susceptibility loci HLA-DRB1, -DQA1 and -DQB1. We identified five DRB1, DQA1 and DQB1 alleles, which were differentially expressed between the C-peptide LOW group on the one hand and the MID or HI group or control group on the other hand (ESM Fig. 1). By contrast, C-peptide MID and HI participants had remarkably similar allele distributions compared with the control group (ESM Fig. 1). We also compared the DRB1-DQA1-DQB1 haplotype frequencies of known type 1 diabetes susceptibility and protection-associated haplotypes between the cohorts. Here, differences were found exclusively between the C-peptide LOW group and the other three groups. Four risk haplotypes (DR3-DQ2, DR4-DQ3, DR7-DQ2 and DR9-DQ2) were identified as being over-represented in participants with LOW C-peptide levels compared with the other C-peptide groups and control participants (Table 3). These haplotypes accounted for almost 50% of all haplotypes observed in this group. One protective haplotype (DR3-DQ4) was under-represented only in the C-peptide LOW group (Table 3). On an individual basis, 65% of participants in the C-peptide LOW group carried at least one risk-associated haplotype, while 1% carried a protective haplotype. These percentages differed significantly from all other subgroups, while there were no differences between C-peptide MID/HI individuals and control individuals (Fig. 2). Taken together, these data suggested that only the C-peptide LOW group carried known type 1 diabetes risk haplotypes and less protective haplotypes, while haplotypes from C-peptide HI and MID individuals resembled those from control individuals.

**Table 3:** HLA haplotype distribution

	Haplotypes	DRB1	DQA1	DQB1	Controls (*n*=266)	HI (*n*=97)	MID (*n*=92)	LOW (*n*=77)	LOW vs Control		LOW vs HI		LOW vs MID	
									*p* value	OR (95% CI)	*p* value	OR (95% CI)	*p* value	OR (95% CI)
Type 1 diabetes risk-associated haplotypes	DR3-DQ2	03:01	05:01	02:01	18	10	7	22	<0.0001	5.06 (2.51, 10.33)	<0.0001	2.8 (1.22, 6.87)	0.0015	3.92 (1.56, 11.23)
	DR4-DQ3^a^			03:02	8	0	4	15	<0.0001	7.5 (2.91, 20.86)	<0.0001	Inf (4.49, Inf)	0.0075	4.52 (1.4, 19.16)
	DR7-DQ2	07:01	03:03	02:01	0	1	0	7			0.0269	8.4 (1.06, 382.2)		
	DR9-DQ2	09:01	03:03	02:01	27	8	6	26	<0.0001	4.05 (2.19, 7.48)	0.0002	4.31 (1.82, 11.41)	<0.0001	5.61 (2.17, 17.19)
Type 1 diabetes protective haplotypes	DR3-DQ4	03:02	04:01	04:02	29	13	16	1	0.0107	0.12 (0, 0.74)	0.0021	0.08 (0, 0.57)	0.0002	0.06 (0, 0.42)

^a^DR4-DQ3 haplotype includes three different combinations: DRB1*04:05 + DQA1*03:03 + DQB1*03:02, DRB1*04:03 + DQA1*03:01 + DQB1*03:02, and DRB1*04:01 + DQA1*03:03 + DQB1*03:02

### Islet cell autoantibodies

**Fig. 3:**
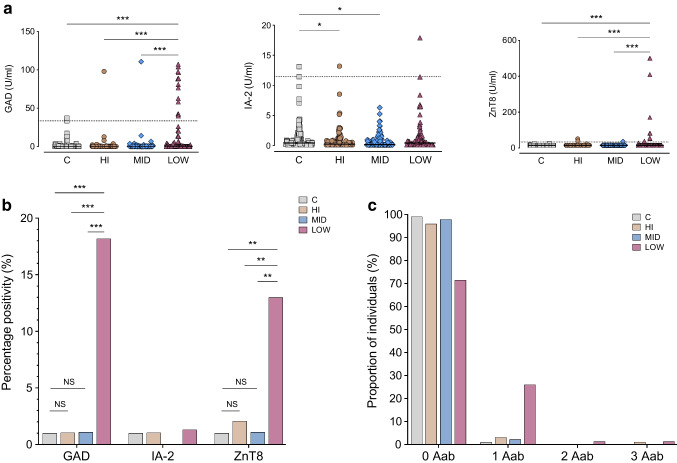
Type 1 diabetes autoantibody positivity. The distribution of GAD, IA2 and ZnT8 autoantibodies in the healthy control group (C) and diabetes subgroups is displayed. (**a**) Quantitative antibody results are depicted as symbol plots with a median line. The dotted line represents the 99th percentile of the control population (*n*=100 healthy control individuals). Statistical significance was determined using the Kruskal–Wallis test followed by Dunn’s post hoc test. (**b**) Bar graphs represent the percentage of individuals testing positive for each autoantibody. (**c**) The proportions of individuals testing negative or positive for one or more autoantibodies. Aab, autoantibody. *p* values below 0.05 were considered significant. *p<0.05; **p<0.01; ***p<0.001

Next, we investigated the prevalence of the GAD, IA-2 and ZnT8 autoantibodies. To find out whether usual cutoffs for autoantibodies developed for European populations can be used in Ghana, we first assessed the quantitative autoantibody results (Fig. 3a). For GAD we found a high background in control participants (range of 0.1 to 37.1 U/ml). IA-2 antibodies in control participants were also very heterogeneously distributed between 0.1 and 13.11 U/ml, and only three individuals with type 1 diabetes had higher IA-2 concentrations. The ZnT8 background in control participants was low; only three control participants had detectable but very low concentrations. Using the cutoffs for GAD, IA-2 and ZnT8 antibodies recommended for Europeans (i.e. 2 U/ml, 2 U/ml and 15 U/ml, respectively), 11%, 18% and 3% of Ghanaian control participants would be classified as autoantibody-positive (ESM Fig. 2). Against this background, we decided to define the cutoffs based on the 99th percentile of the local control cohort as generally recommended [7] (i.e. ≥37.1 U/ml for GAD, ≥13.11 U/ml for IA-2 and ≥24.56 U/ml for ZnT8).

Using these new cutoffs, we compared autoantibody positivity between the C-peptide groups. While no differences were seen for IA-2, elevated proportions of GAD and ZnT8 were exclusively found in the C-peptide LOW group compared with all other groups (18.2% and 13%, respectively) (Fig. 3b). There was no difference between C-peptide MID/HI and control participants. Analysing simultaneous autoantibody occurrence in individuals, we found that even in the C-peptide LOW cohort, most individuals (71%) lacked detectable autoantibodies. Only 26%, 1% and 1% had detectable one, two or three autoantibodies, respectively (Fig. 3c).

As islet autoantibodies are known to decrease after diagnosis, we included autoantibody measurements in a small cohort (*n*=54) of individuals clinically diagnosed with type 1 diabetes less than 1 year ago. While no autoantibodies could be detected in the new-onset C-peptide HI group, elevated proportions of GAD and ZnT8 were found in both the C-peptide MID and LOW new-onset cohorts (ESM Fig. 3). However, a significant portion of participants in the new-onset C-peptide LOW and MID cohorts (50% and 75%, respectively) tested negative for autoantibodies. Overall, these data showed a low proportion of positive autoantibodies in new-onset and long-standing type 1 diabetes, with the highest positivity in the C-peptide LOW groups.

### Evaluation of clinical subgroups with retained insulin secretion

Against the background of clinical heterogeneity in the C-peptide MID and HI cohort, we further compared groups of individuals with different clinical presentations for their autoimmune profiles. When comparing underweight, normal, and overweight/obese participants in the combined C-peptide MID/HI group, we found no differences in HLA risk haplotypes or autoantibodies (ESM Table 2). Similarly, there were no differences between groups with or without DKA/ketosis (ESM Table 3). To further assess a potential honeymoon period’s influence, we repeated the comparisons of the C-peptide groups in a subcohort with a disease course of over 3 years, which yielded similar results (ESM Fig. 4).

### Non-autoimmune pathways

**Fig. 4:**
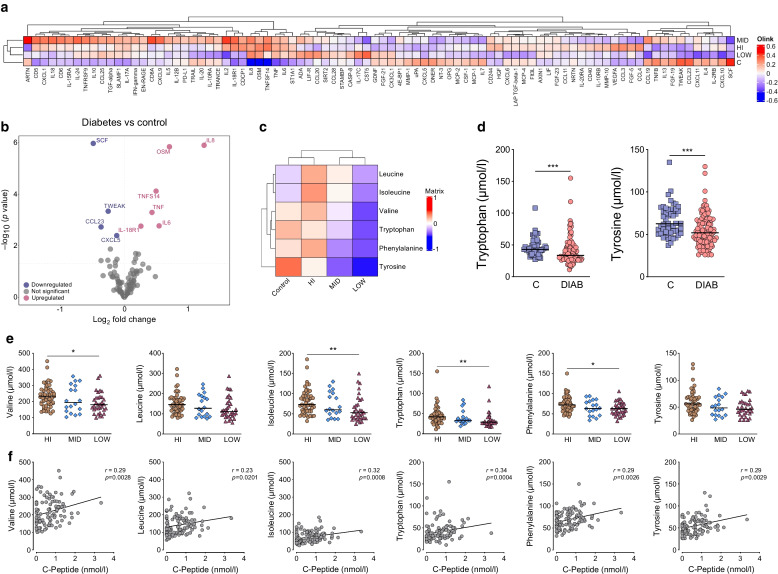
Inflammation-related protein and amino acid profiling. Analysis of Olink inflammatory-related proteins (IRPs) and six branched-chain and aromatic amino acids in healthy control (C; *n*=58) and diabetes subgroups (*n*=118; HI *n*=59, MID *n*=20, LOW *n*=39) was performed. (**a**) Heatmap displaying the relative expression levels of IRPs across study groups. Colours represent *z* scores, with red indicating higher expression and blue indicating lower expression. Hierarchical clustering was performed on rows (study groups) and columns (proteins). (**b**) Volcano plot comparing protein expression between healthy control participants and individuals with diabetes combined. The *x*-axis represents the log_2_ fold change, and the y-axis represents the −log_10_ *p* value, with the threshold (unadjusted *p* value set at 0.05) indicated with dashed lines. Red dots indicate significantly upregulated proteins, and blue dots indicate significantly downregulated proteins in the diabetes group. Grey dots represent proteins with no significant change. Only proteins that are significantly differentially expressed above the significance threshold (adjusted *p* value) are named and marked. (**c**) Heatmap illustrating the relative levels of six amino acids across healthy control and diabetes subgroups. Colours represent *z* scores, with red indicating higher levels and blue indicating lower levels. Hierarchical clustering was performed on both rows (amino acids) and columns (study groups). (**d**) Symbol plots showing the expression levels of significant amino acids between control participants and all people with diabetes. Individual points are shown with the median as a straight line. (**e**) Symbol plots showing the expression levels of amino acids across the diabetes subgroups. Individual points are shown with the median as a straight line. (**f**) Scatter plots showing the correlation between each of the six amino acid metabolites and C-peptide levels across all individuals with diabetes. A solid line in each plot represents the linear regression fit. Spearman correlation coefficients (*r*) and *p* values are provided for each correlation. Multiple testing corrections of panels (**a**), (**b**) and (**c**) were applied using the Benjamini–Hochberg method. The Kruskal–Wallis test was performed for group comparisons in (**e**), and Dunn’s correction was applied for multiple comparisons. Statistically significant differences between groups are indicated. *p<0.05; **p<0.01; ***p<0.001

To further assess non-autoimmune pathways, we investigated whether systemic inflammation or metabolic dysregulation differed between the C-peptide groups by analysing serum proinflammatory cytokines and amino acids. To avoid an age- and sex-related bias in the investigation of non-autoimmune pathways, we selected subcohorts of control participants, C-peptide HI, MID and LOW participants, who were similar in age and sex (total *n*=176, subcohort characteristics in ESM Table 4). Serum protein expression was visualised in a heatmap (Fig. 4a). After correction for multiple comparisons, 10 proteins were differentially expressed between the diabetes and control groups. While six proteins (oncostatin-M (OSM), TNF ligand superfamily member 14 (TNFSF14), TNF, IL-6, IL-8, and IL-18R) were higher in the diabetes group, four proteins were lower in diabetes compared with control participants (stem cell factor (SCF), TNF-like weak inducer of apoptosis (TWEAK), C-C motif chemokine 23 (CCL23), C-X-C motif chemokine 5 (CXCL5); Fig. 4b). However, no differences were found across C-peptide HI, MID and LOW groups (ESM Fig. 5). The concentrations of branched-chain (valine, leucine and isoleucine) and aromatic amino acids (tryptophan, phenylalanine and tyrosine) were also visualised as a heatmap in Fig. 4c. Only tryptophan and tyrosine significantly differed between the control group and the combined diabetes group with lower levels in diabetes (Fig. 4d). When the diabetes subgroups were compared, the C-peptide HI cohort had higher levels of valine, isoleucine, tryptophan and phenylalanine than the LOW group (Fig. 4e). To investigate whether the amino acid concentrations were associated with the residual insulin secretion capacity, we correlated amino acid levels with absolute C-peptide concentrations (Fig. 4f). Remarkably, we found significant positive correlations between all aromatic and branched-chain amino acids and the C-peptide level (Fig. 4f). Taken together, we discovered that aromatic and branched-chain amino acids varied between C-peptide groups, suggesting a possible connection between the amino acid profile and the individual’s preserved insulin production.

## Discussion

In this study, individuals with clinically diagnosed type 1 diabetes from Ghana were categorised based on their serum C-peptide levels. Less than one-third of participants had low C-peptide levels (<0.2 nmol/l) that would be expected for long-term type 1 diabetes. Only this C-peptide LOW group exhibited typical type 1 diabetes characteristics, including an association with HLA class II risk haplotypes and increased type 1 diabetes autoantibodies. Conversely, the C-peptide MID and HI group showed similarities to the control group in terms of protective HLA haplotypes and the absence of autoantibodies, suggesting that they do not have autoimmune type 1 diabetes. Misclassification of diabetes is common and even more difficult in sub-Saharan Africa where atypical diabetes presentations such as malnutrition-related diabetes, KPD and type 2 diabetes in the absence of overweight and obesity have been described [1, 4–6]. It can only be speculated which pathology underlies the diabetes diagnoses from the C-peptide MID and HI groups. Since all participants had been clinically diagnosed with type 1 diabetes, as evidenced by their clinical presentation with symptoms such as ketosis or DKA, KPD could be a possible underlying condition. According to the recent WHO classification, KPD is a non-immune ketosis-prone form of type 2 diabetes that has high ketone production and severe insulin deficiency at onset [24]. It typically occurs in people of African, Hispanic or Asian ancestry. Individuals with KPD often regain beta cell function and can be discontinued from insulin later. Our data indicated a prevalence of either ketosis or DKA at onset in 47% and 57% of the C-peptide MID and HI groups. However, the purely anamnestic survey suggests that these numbers may be underestimated, rendering KPD a significant underlying form of diabetes. Nevertheless, other forms of diabetes can contribute to misclassification. The biometric data of the C-peptide MID and HI group, with an underweight proportion of only 11% and 5%, respectively, suggests that malnutrition-associated forms of diabetes are unlikely to account for a significant portion of misclassification. By contrast, the high prevalence of overweight and obesity, together with an older age at onset, would align with the characteristics of both classic type 2 diabetes and KPD. However, the fact that all participants of our cohort were long-term insulin dependent and clinically presented like type 1 diabetes makes the diagnosis of classic type 2 diabetes less probable compared with KPD.

HLA class II typing revealed a distinct genetic association in the C-peptide LOW group compared with the MID and HI groups, which resembled those of the control group. Four previously described type 1 diabetes susceptibility haplotypes were over-represented only in the C-peptide LOW group, while a protective haplotype was diminished. Some of these haplotypes, such as the highly susceptible DR3-DQ2 and DR4-DQ3, have been well-described in European type 1 diabetes cohorts [11]. Others, like the risk-associated DR7-DQ2 and DR9-DQ2, and the protective associated DR3-DQ4 haplotype, have been mentioned in African type 1 diabetes studies [25, 26]. This study found no significant HLA class II risk or susceptibility association in the C-peptide MID and HI groups. This finding is consistent with the few existing studies on individuals with KPD that did not detect any HLA class II haplotype associations [27]. Data on genetic susceptibility to type 2 diabetes would be valuable in further assessing the role of classic type 2 diabetes in misclassification. However, these data were beyond the scope of this study.

Presence of type 1 diabetes autoantibodies is a hallmark of type 1 diabetes and aids classification. Most studies on KPD report a lack of autoantibodies or insulin- or islet-specific immunoreactivity, with a small number of exceptions [28, 29]. Accordingly, only in the C-peptide LOW long-term, but not in the C-peptide MID and HI group, increased rates of GAD and ZnT8 (but not IA-2) were detected. However, the proportions of these autoantibodies were only low, which aligns with studies that have reported lower rates of autoantibody positivity in type 1 diabetes in sub-Saharan Africa [reviewed in 1]. Antibody proportions were moderately higher in the new-onset cohort and higher percentages of GAD and ZnT8 positivity were also observed in the C-peptide MID new-onset group, suggesting that the chosen C-peptide cutoffs may not be optimal within the first year after onset.

Systemic low-grade inflammation is frequently observed in diabetes but differs between diabetes types [12]. We found 10 differentially expressed inflammatory serum proteins between control participants and individuals with diabetes. The proteins that were increased in diabetes, including OSM, IL-8, IL-18R, TNFSF14, TNF and IL-6, play roles in obesity [30–32], insulin resistance [33, 34], beta cell function [35] and vascular diabetes complications [36, 37]. However, none of these proteins showed differences between the C-peptide groups. When analysing the amino acid profile of branched-chain and aromatic amino acids, we found significant differences between the C-peptide HI and LOW groups and a positive correlation between branched-chain and aromatic amino acids and C-peptide levels. This is in accordance with previous studies that suggested an association between these amino acids and insulin resistance [14]. In KPD, amino acids might play an additional role, as reported that amino and fatty acid metabolic deteriorations contribute to the ketosis proneness [38]. Further studies in larger cohorts are required to unravel these pathologies.

Despite its contributions, this study has limitations. We matched diabetes and control groups by age and sex, but there were differences in age, sex and BMI between the C-peptide groups, which may affect further results. To mitigate this, we selected subcohorts for the analysis of non-autoimmune pathways that did not differ in age and sex distribution, but BMI differences were still found, potentially impacting results. There was no record of the specific fasting status, and no concurrent blood glucose levels were measured.

This study identified great heterogeneity in clinically diagnosed type 1 diabetes in Ghana. Approximately two-thirds of all participants exhibited maintained C-peptide levels, lack the typical type 1 diabetes genetic markers and autoantibodies and may therefore be miscategorised. The maintained C-peptide levels indicate that some of these individuals may be able to manage their condition without insulin. In previous studies, over 75% of individuals with KPD could be discontinued from insulin therapy early after diagnosis, typically within 14 weeks, particularly those with high C-peptide levels [39]. Careful re-evaluation of the treatment regimen for the C-peptide MID group, and particularly the C-peptide HI group, is therefore essential to ensure an optimal care strategy. Beyond that, it would be desirable to establish clear diagnostic criteria to improve classification accuracy at diagnosis. This could potentially not only optimise individual outcomes but also yield economic benefits through reduced healthcare costs associated with prolonged insulin therapy.

Consequently, this study advocates for a shift in how diabetes is understood and managed in Ghana, as well as in other settings with a high proportion of individuals who present with atypical diabetes forms. This endeavour would ultimately contribute to optimising patient care and enhancing overall health outcomes across the population.

## Supplementary Information

Below is the link to the electronic supplementary material.

ESM (PDF 600 KB)

## Data Availability

The datasets generated during and/or analysed in the current study are available from the corresponding author upon reasonable request.

## Abbreviations

DKA: Diabetic ketoacidosis
KATH: Komfo Anokye Teaching Hospital
KPD: Ketosis-prone type 2 diabetes
OSM: Oncostatin-M
TNFSF14: TNF ligand superfamily member 14

## References

[1] Katte JC, McDonald TJ, Sobngwi E, Jones AG (2023) The phenotype of type 1 diabetes in sub-Saharan Africa. Front Public Health 11:1014626 36778553 10.3389/fpubh.2023.1014626 PMC9912986

[2] Seyfarth J, Sarfo-Kantanka O, Rosenbauer J, Phillps RO, Jacobsen M (2019) Type-1 diabetes onset age and sex differences between Ghanaian and German urban populations. J Diabetes 11(12):1002–1004 31420945 10.1111/1753-0407.12978

[3] Godman B, Basu D, Pillay Y et al (2020) Ongoing and planned activities to improve the management of patients with Type 1 diabetes across Africa; implications for the future. Hosp Pract (1995) 48:51–67 32196395 10.1080/21548331.2020.1745509

[4] Gill GV, Mbanya JC, Ramaiya KL, Tesfaye S (2009) A sub-Saharan African perspective of diabetes. Diabetologia 52:8–16 18846363 10.1007/s00125-008-1167-9

[5] Mbanya JC, Motala AA, Sobngwi E, Assah FK, Enoru ST (2010) Diabetes in sub-Saharan Africa. Lancet (London, England) 375:2254–2266 20609971 10.1016/S0140-6736(10)60550-8

[6] Gujral UP, Weber MB, Staimez LR, Narayan KMV (2018) Diabetes among non-overweight individuals: an emerging public health challenge. Curr Diab Rep 18:60 29974263 10.1007/s11892-018-1017-1

[7] Bingley PJ (2010) Clinical applications of diabetes antibody testing. J Clin Endocrinol Metab 95:25–33 19875480 10.1210/jc.2009-1365

[8] Jones AG, Hattersley AT (2013) The clinical utility of C-peptide measurement in the care of patients with diabetes. Diabetic Med J Br Diabetic Assoc 30:803–817 10.1111/dme.12159 PMC3748788 23413806

[9] Foteinopoulou E, Clarke CAL, Pattenden RJ et al (2021) Impact of routine clinic measurement of serum C-peptide in people with a clinician-diagnosis of type 1 diabetes. Diabetic Med J Br Diabetic Assoc 38:e14449 10.1111/dme.14449 33131101

[10] Sarfo-Kantanka O, Asamoah-Boaheng M, Arthur J et al (2020) Trends in type 1 diabetes diagnosis in Ghana. Int Health 14(4):442–446 10.1093/inthealth/ihz096 PMC9248061 32050027

[11] Noble JA, Erlich HA (2012) Genetics of type 1 diabetes. Cold Spring Harb Perspect Med 2:a007732 22315720 10.1101/cshperspect.a007732 PMC3253030

[12] Castelblanco E, Hernández M, Castelblanco A et al (2018) Low-grade inflammatory marker profile may help to differentiate patients with LADA, classic adult-onset type 1 diabetes, and type 2 diabetes. Diabetes Care 41:862–868 29358494 10.2337/dc17-1662

[13] Rohm TV, Meier DT, Olefsky JM, Donath MY (2022) Inflammation in obesity, diabetes, and related disorders. Immunity 55:31–55 35021057 10.1016/j.immuni.2021.12.013 PMC8773457

[14] Würtz P, Soininen P, Kangas AJ et al (2013) Branched-chain and aromatic amino acids are predictors of insulin resistance in young adults. Diabetes Care 36:648–655 23129134 10.2337/dc12-0895 PMC3579331

[15] Arneth B, Arneth R, Shams M (2019) Metabolomics of type 1 and type 2 diabetes. Int J Mol Sci 20:2467 31109071 10.3390/ijms20102467 PMC6566263

[16] Apostolopoulou M, Lambadiari V, Roden M, Dimitriadis GD (2025) Insulin resistance in type 1 diabetes: pathophysiological, clinical, and therapeutic relevance. Endocr Rev 46:317–348 39998445 10.1210/endrev/bnae032 PMC12063105

[17] (2024) 2. Diagnosis and classification of diabetes: standards of care in diabetes-2024. Diabetes Care 47:S20–s42 10.2337/dc24-S002 PMC10725812 38078589

[18] U.S. Centers for Disease Control and Prevention (CDC) Child and Teen BMI Calculator, U.S. Centers for Disease Control and Prevention (CDC). Available from https://www.cdc.gov/bmi/child-teen-calculator/index.html. Accessed 6 Feb 2025

[19] Berger B, Stenström G, Sundkvist G (2000) Random C-peptide in the classification of diabetes. Scand J Clin Lab Invest 60:687–693 11218151 10.1080/00365510050216411

[20] Holt RIG, DeVries JH, Hess-Fischl A et al (2021) The management of type 1 diabetes in adults. A consensus report by the American Diabetes Association (ADA) and the European Association for the Study of Diabetes (EASD). Diabetes Care 44:2589–2625 34593612 10.2337/dci21-0043

[21] Enczmann J, Balz V, Hoffmann M et al (2021) Next generation sequencing identifies the HLA-DQA1*03:03 allele in the type 1 diabetes risk-associated HLA-DQ8 serotype. Genes (Basel) 12:1879 34946827 10.3390/genes12121879 PMC8701008

[22] Zaharia OP, Strassburger K, Strom A et al (2019) Risk of diabetes-associated diseases in subgroups of patients with recent-onset diabetes: a 5-year follow-up study. Lancet Diabetes Endocrinol 7:684–694 31345776 10.1016/S2213-8587(19)30187-1

[23] Prinsen HCMT, Schiebergen-Bronkhorst BGM, Roeleveld MW et al (2016) Rapid quantification of underivatized amino acids in plasma by hydrophilic interaction liquid chromatography (HILIC) coupled with tandem mass-spectrometry. J Inherit Metab Dis 39:651–660 27099181 10.1007/s10545-016-9935-z PMC4987396

[24] World Health Organization Licence: CC BY-NC-SA 3.0 IGO (2019) Classification of diabetes mellitus. Available from https://iris.who.int/handle/10665/325182. Accessed 6 Feb 2025

[25] Noble JA, Johnson J, Lane JA, Valdes AM (2013) HLA class II genotyping of African American type 1 diabetic patients reveals associations unique to African haplotypes. Diabetes 62:3292–3299 23801574 10.2337/db13-0094 PMC3749336

[26] Noble JA, Johnson J, Lane JA, Valdes AM (2011) Race-specific type 1 diabetes risk of HLA-DR7 haplotypes. Tissue Antigens 78:348–351 21988721 10.1111/j.1399-0039.2011.01772.x PMC3193161

[27] Lebovitz HE, Banerji MA (2018) Ketosis-prone diabetes (Flatbush Diabetes): an emerging worldwide clinically important entity. Curr Diab Rep 18:120 30280274 10.1007/s11892-018-1075-4 PMC6182625

[28] Maldonado M, Hampe CS, Gaur LK et al (2003) Ketosis-prone diabetes: dissection of a heterogeneous syndrome using an immunogenetic and beta-cell functional classification, prospective analysis, and clinical outcomes. J Clin Endocrinol Metab 88:5090–5098 14602731 10.1210/jc.2003-030180

[29] Brooks-Worrell BM, Iyer D, Coraza I et al (2013) Islet-specific T-cell responses and proinflammatory monocytes define subtypes of autoantibody-negative ketosis-prone diabetes. Diabetes Care 36:4098–4103 24130366 10.2337/dc12-2328 PMC3836121

[30] Sanchez-Infantes D, White UA, Elks CM et al (2014) Oncostatin m is produced in adipose tissue and is regulated in conditions of obesity and type 2 diabetes. J Clin Endocrinol Metab 99:E217-225 24297795 10.1210/jc.2013-3555 PMC3913819

[31] Safabakhsh D, Jazaeri M, Abdolsamadi H, Abassi E, Farhadian M (2022) Comparison of salivary interleukin-6, interleukin-8, C - reactive protein levels and total antioxidants capacity of obese individuals with normal-weight ones. Rom J Intern Med 60:215–221 35976790 10.2478/rjim-2022-0013

[32] Dandona P, Ghanim H, Monte SV et al (2014) Increase in the mediators of asthma in obesity and obesity with type 2 diabetes: reduction with weight loss. Obesity (Silver Spring) 22:356–362 23804543 10.1002/oby.20524

[33] Piquer-Garcia I, Campderros L, Taxerås SD et al (2020) A Role for Oncostatin M in the Impairment of Glucose Homeostasis in Obesity. J Clin Endocrinol Metab 105:e337-348 31606738 10.1210/clinem/dgz090 PMC7112982

[34] Akash MSH, Rehman K, Liaqat A (2018) Tumor necrosis factor-alpha: role in development of insulin resistance and pathogenesis of type 2 diabetes mellitus. J Cell Biochem 119:105–110 28569437 10.1002/jcb.26174

[35] Dludla PV, Mabhida SE, Ziqubu K et al (2023) Pancreatic β-cell dysfunction in type 2 diabetes: Implications of inflammation and oxidative stress. World J Diabetes 14:130–146 37035220 10.4239/wjd.v14.i3.130 PMC10075035

[36] Ikeda S, Sato K, Takeda M et al (2021) Oncostatin M is a novel biomarker for coronary artery disease - A possibility as a screening tool of silent myocardial ischemia for diabetes mellitus. Int J Cardiol Heart Vasc 35:100829 34235245 10.1016/j.ijcha.2021.100829 PMC8250159

[37] Cimini FA, Barchetta I, Porzia A et al (2017) Circulating IL-8 levels are increased in patients with type 2 diabetes and associated with worse inflammatory and cardiometabolic profile. Acta Diabetol 54:961–967 28836077 10.1007/s00592-017-1039-1

[38] Patel SG, Hsu JW, Jahoor F et al (2013) Pathogenesis of A(-)beta(+) ketosis-prone diabetes. Diabetes 62:912–922 23160531 10.2337/db12-0624 PMC3581228

[39] Mauvais-Jarvis F, Sobngwi E, Porcher R et al (2004) Ketosis-prone type 2 diabetes in patients of sub-Saharan African origin: clinical pathophysiology and natural history of beta-cell dysfunction and insulin resistance. Diabetes 53:645–653 14988248 10.2337/diabetes.53.3.645

