# Supplementary material for: Heterogeneity in clinically diagnosed type 1 diabetes: characterising a unique cohort with maintained C-peptide secretion in Ghana
Source: Diabetologia. 2025 Nov 1;69(2):308–20. doi: 10.1007/s00125-025-06576-3 (PMC12779725; doi:10.1007/s00125-025-06576-3)
Supplement: Supplementary file 1 — ESM (PDF 600 KB) [file 125_2025_6576_MOESM1_ESM.pdf]

ESM Table 1. Clinical characteristics of the diabetes new-onset study participants

	**hi**	**mid**	**low**	*p-value*
Number, *n*	20	20	14	
Female/male (*% female*)	15/5 (75%)	11/9 (55%)	6/8 (43%)	0.18
Age (*median, IQR*)	15 (12-44)	15 (9-69)	14.5 (12-58)	0.44
HbA1c [mmol/mol] (IQR)	62 (40-98)	74 (59-99)	96 (72-125)	0.05
HbA1c [%] (IQR)	7.9 (6-11)	8.9 (8-11)	11 (9-14)	
BMI (*kg/m^2*)	24.9 (20-38)	18.7 (16-25)	17.9 (16-31)	0.01
BMI classification (*n*)				
underweight	3 (15%)	10 (50%)	8 (57%)	
normal	7 (35%)	10 (50%)	4 (29%)	
overweight	5 (25%)	0 (0%)	1 (7%)	
obese	5 (25%)	0 (0%)	1 (7%)	
Presentation at onset (n)				0.44
DKA	7 (35%)	6 (30%)	7 (50%)	
Ketosis	6 (30%)	3 (15%)	3 (21%)	
hyperglycaemia	7 (35%)	8 (40%)	3 (21%)	
By screening	0 (0%)	3 (15%)	1 (7%)	

Data are presented as median and interquartile range (IQR) unless otherwise specified. BMI classification and Presentation at onset are shown as number of cases (%). Categorical variables were compared using Fischer's exact test, and continuous variables using one-way ANOVA, as appropriate. Statistical significance was defined as $p < 0.05$.

ESM Table 2. Clinical characteristics of the diabetes cohort stratified by BMI

	**Underweight (HI & MID)**	**Normal (HI & MID)**	**Overweight + Obese (HI & MID)**	*p-value*
Number, *n*	15	69	105	
Age at onset (*median, IQR*)	14 (11-21)	28 (17-43)	35 (25-48)	<0.001
At least 1 HLA risk allele (*n*)	1 (7%)	9 (13%)	22 (21%)	0.27
Autoantibodies (*n*)				0.15
0	14 (93%)	65 (94%)	104 (99%)	
1	1 (7%)	3 (4%)	1 (1%)	
2	0 (0%)	0 (0%)	0 (0%)	
3	0 (0%)	1 (2%)	0 (0%)	
Presentation at onset (*n*)				0.54
DKA	8 (53%)	22 (32%)	31 (30%)	
Ketosis	1 (7%)	12 (17%)	18 (17%)	
hyperglycaemia	4 (27%)	29 (42%)	43 (41%)	
By screening	0 (0%)	1 (2%)	5 (5%)	

Data are presented as median and interquartile range (IQR) unless otherwise specified. Categorical variables were compared using Fischer's exact test, and continuous variables using one-way ANOVA, as appropriate. Statistical significance was defined as $p < 0.05$.

ESM Table 3. Clinical characteristics of the diabetes cohort stratified by their presentation at onset

	**Combined DKA/Ketosis (HI & MID)**	**Non ketoacidosis/ketosis (HI & MID)**	*p-value*
Number, *n*	92	85	
Age at onset (*median, IQR*)	30 (16-45)	33 (21-45)	0.23
At least 1 HLA risk allele (*n*)	16 (17.4%)	19 (22.4%)	0.45
Autoantibodies (*n*)			0.51
0	90 (98%)	81 (95%)	
1	2 (2%)	3 (4%)	
2	0 (0%)	0 (0%)	
3	0 (0%)	1 (1%)	
BMI (kg/m^2)	26 (22-29)	26 (22-30)	0.36

Data are presented as median and interquartile range (IQR) unless otherwise specified. Autoantibody numbers are shown as number of cases (%). Categorical variables were compared using Fischer's exact test, and continuous variables using Student's t-test, as appropriate. Statistical significance was defined as $p < 0.05$.

ESM Table 4. Clinical characteristics of the omics subcohort

	**Control**	**T1D**		*p-value*
Number, *n*	58	118		
Female/male (*% female*)	40/18 (68%)	82/36 (69%)		>0.99
Age (*median, IQR*)	36 (21-49)	35 (21-49)		0.98
	**Diabetes subgroups**			
	HI	MID	LOW	
Number, *n*	59	20	39	
Female/male (*% female*)	41/18 (69%)	17/3 (85%)	24/15 (62%)	0.18
Age [years] (*IQR*)	44 (21-52)	23 (19-38)	30 (23-45)	0.06
BMI classification [number]				
underweight	5 (9%)	1 (5%)	5 (13%)	
normal	20 (34%)	10 (50%)	24 (62%)	
overweight	18 (31%)	4 (20%)	5 (13%)	
obese	16 (27%)	5 (25%)	5 (13%)	
BMI (kg/m^2)	26 (23-31)	20 (18-23)	20 (18-23)	<0.001
HbA1c [mmol/mol] (*IQR*)	79.9 (60-148)	92 (70-123)	92 (63-132)	0.06
HbA1c [%] (*IQR*)	9.5 (7.6-10.9)	9.9 (8.8-12)	10.1 (7.6-12.5)	

Data are presented as median and interquartile range (IQR) unless otherwise specified. BMI classification is shown as number of cases (%). Categorical variables were compared using Fischer's exact test, and continuous variables using one-way ANOVA, as appropriate. Statistical significance was defined as $p < 0.05$.

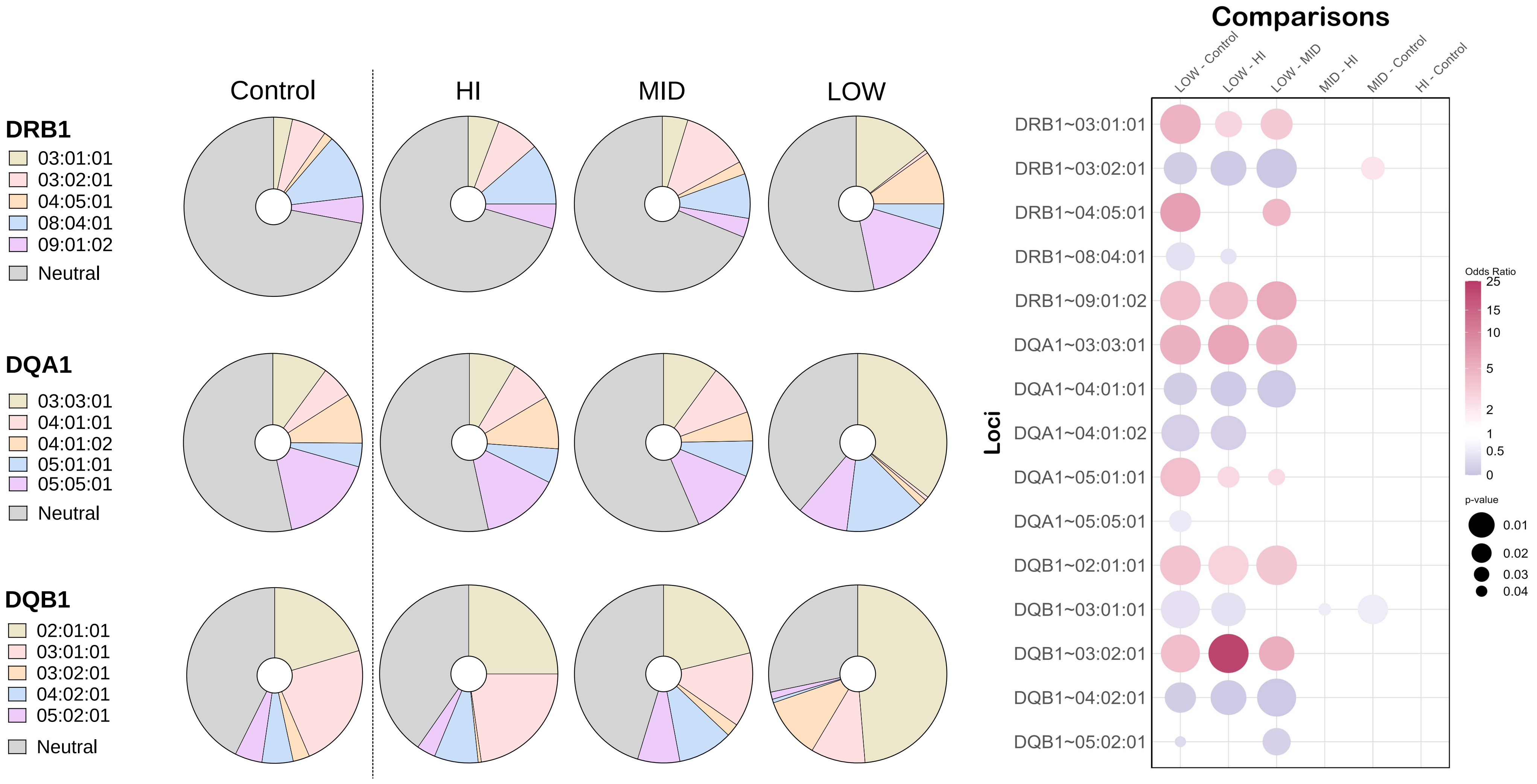

ESM Figure 1. ***HLA-DRB1, -DQA1, and -DQB1 allele distribution.*** Pie charts depict the distribution of HLA-DRB1, -DQA1, and -DQB1 alleles within study groups. HLA-DRB1, -DQA1, and -DQB1 loci across all four groups are shown. Each pie chart represents one locus in one group, with slices showing the proportion of each allele that displays significant differences between groups. The bubble chart on the right displays pairwise comparisons of allele distributions between groups. The x-axis represents the comparison pairs, and the y-axis lists the significant alleles. Bubble color indicates the odds ratio (OR) with a gradient from blue (OR < 1) to red (OR > 1). Bubble size represents the p-value, with larger bubbles indicating lower p-values (higher significance). Significant differences in HLA alleles were calculated using the chi-square ($\chi 2$) test. Only comparisons with significant p-values (below 0.05) are depicted

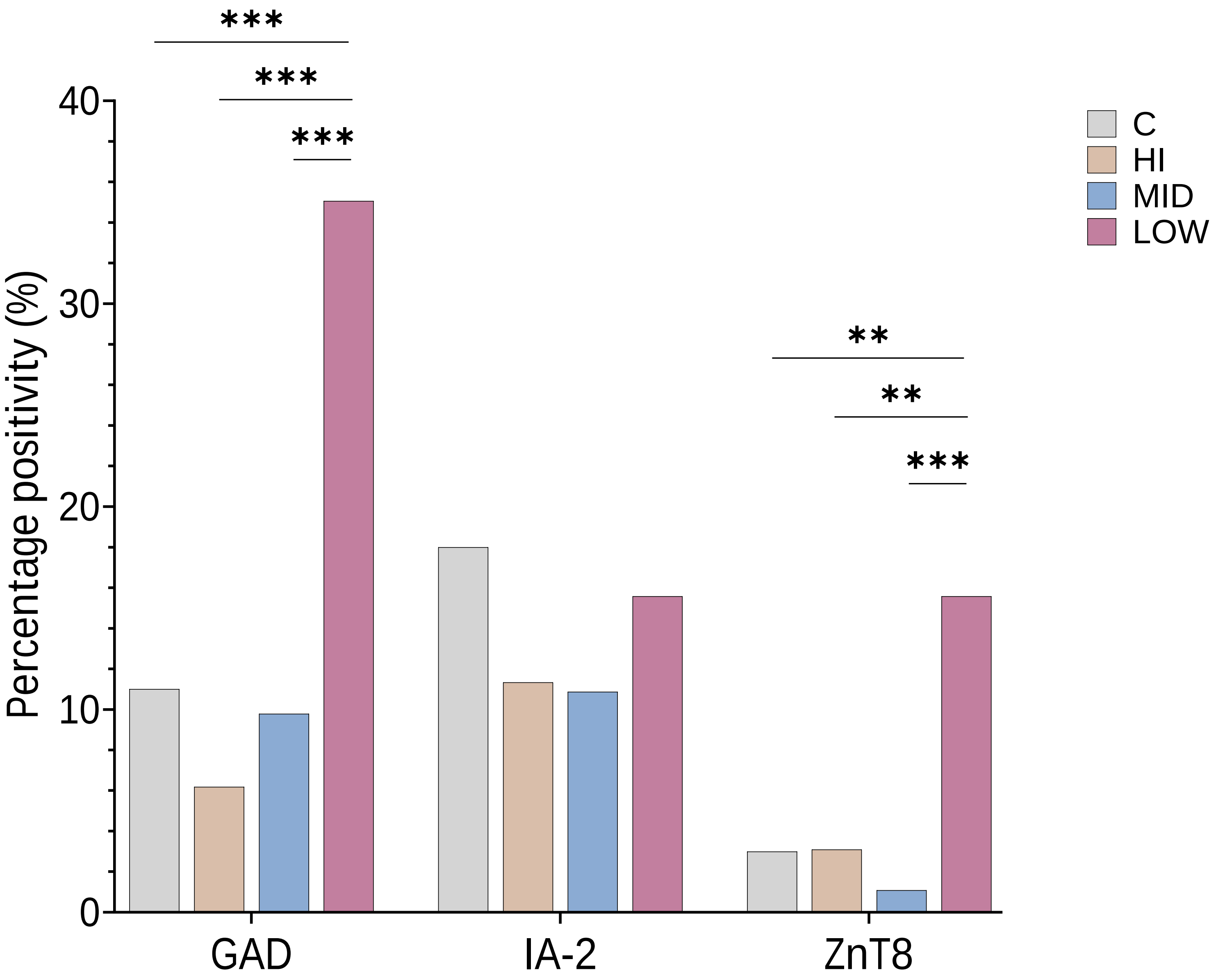

ESM Figure 2. ***Type 1 diabetes autoantibody positivity (uncorrected).*** The distribution of GAD, IA2, and ZnT8 autoantibodies in healthy controls and long-term diabetes subgroups is displayed. This depiction uses the cutoffs for GAD, IA-2, and ZnT8 antibodies recommended for Europeans (i.e., 2 U/ml, 2 U/ml, and 15 U/ml, respectively). Bar graphs represent the percentage of individuals testing positive for each autoantibody. P-values below 0.05 were considered significant. P-values are indicated as asterisks with *, p<0.05; **, p<0.01; ***, p<0.001

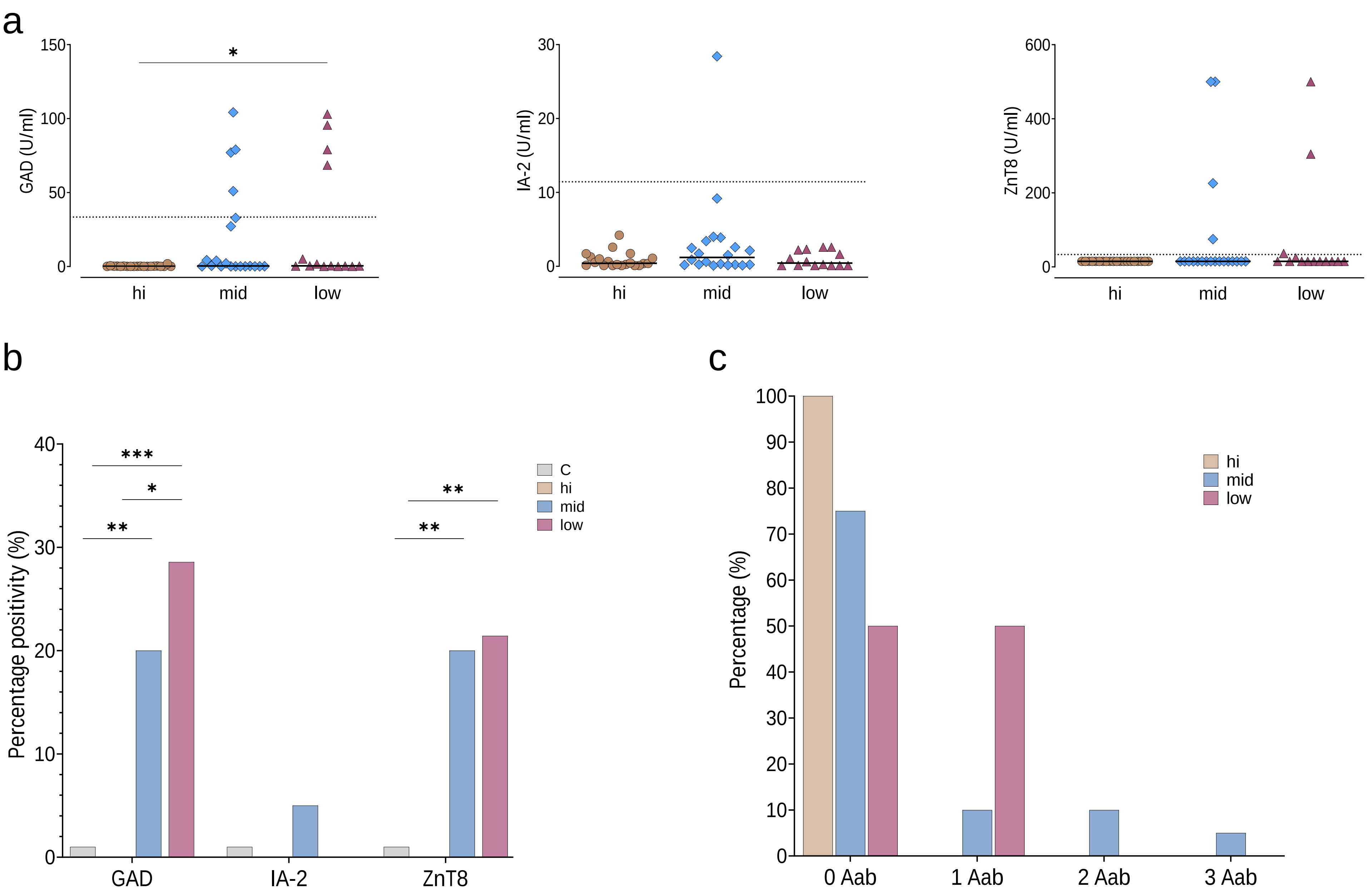

ESM Figure 3. ***Type 1 diabetes autoantibody positivity in new-onset patients*** The distribution of GAD, IA2, and ZnT8 autoantibodies in healthy controls and new-onset diabetes subgroups is displayed. (a) Quantitative antibody results are depicted as symbol plots with a median line. The dotted line represents the 99th percentile of the control population (n=100 healthy controls). Statistical significance was determined using the Kruskal-Wallis test followed by Dunn's post-hoc test. (b) Bar graphs represent the percentage of individuals testing positive for each autoantibody. (c) The proportions of individuals testing negative or positive for one or more autoantibodies. Autoantibody (Aab). P-values below 0.05 were considered significant. P-values are indicated as asterisks with *, $p<0.05$; **, $p<0.01$; ***, $p<0.001$

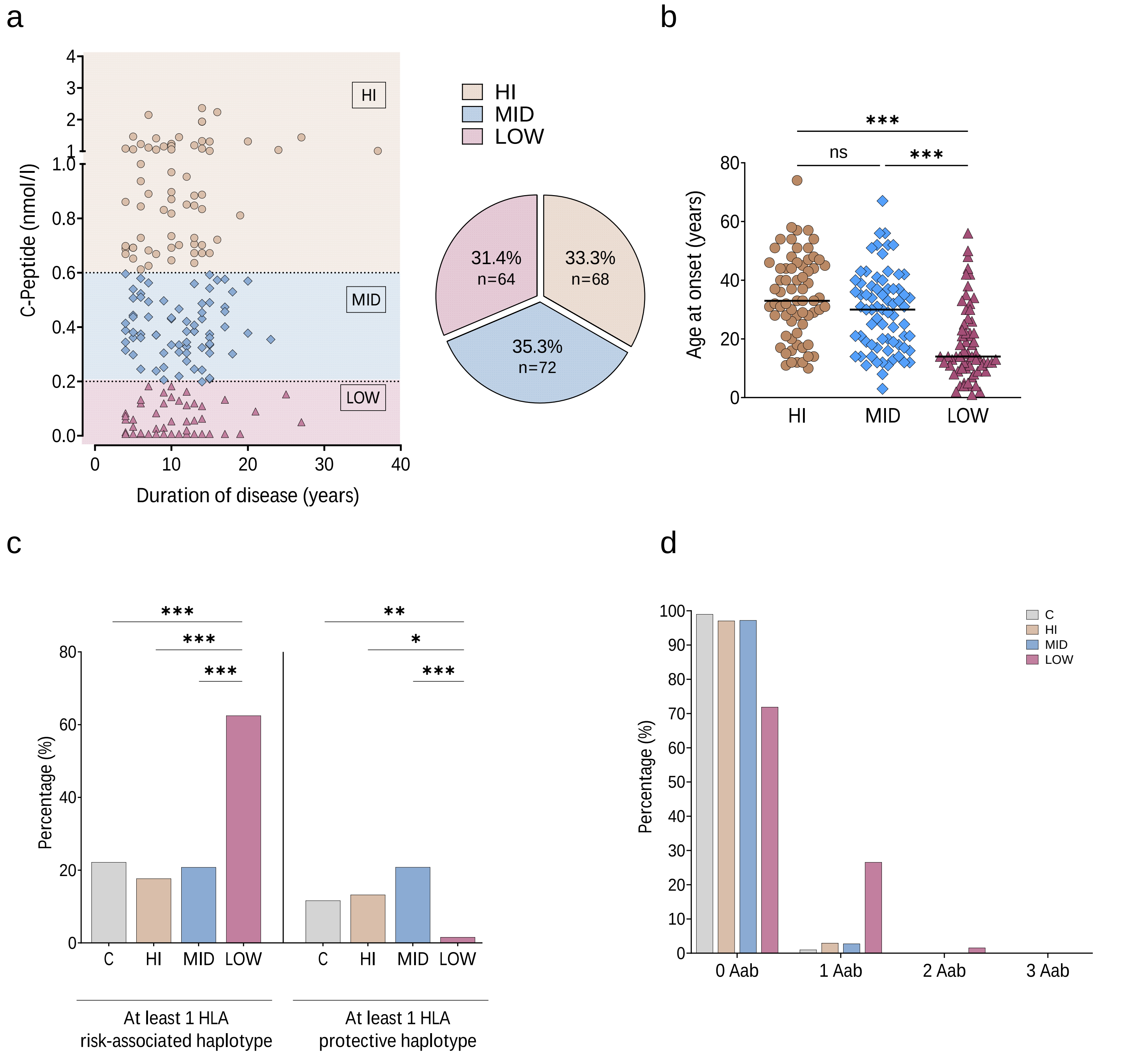

ESM Figure 4. ***Clinical characteristics of diabetes subgroups with > 3-year disease duration.*** Long-term diabetes subgroups were recalculated with a >3-year disease duration cutoff instead of > 1 year. (a) Distribution of individuals with diabetes into subgroups using C-peptide values (cut-offs 0.2 and 0.6 nmol/l) plotted against the duration of diabetes. Proportions and absolute numbers in diabetes subgroups (C-peptide HI, MID, and LOW) are depicted as a pie chart. (b) Age at diabetes onset in diabetes subgroups is depicted. (c) The percentages of individuals carrying at least one risk-associated or protective HLA class II haplotype in the diabetes subgroups and controls are represented by bar graphs. (d) The proportions of individuals testing negative or positive for one or more autoantibodies. Autoantibody (Aab). Symbol plots are depicted with a median line. Differentiation by color and shape plots is indicated: HI (yellow circles), MID (blue diamonds), and LOW (red triangles). The chi-square ($\chi 2$) test was applied to categorical parameters, and the Kruskal-Wallis test was used for continuous variables. P-values below 0.05 were considered significant. P-values are indicated as asterisks with *, p<0.05; **, p<0.01; ***, p<0.001

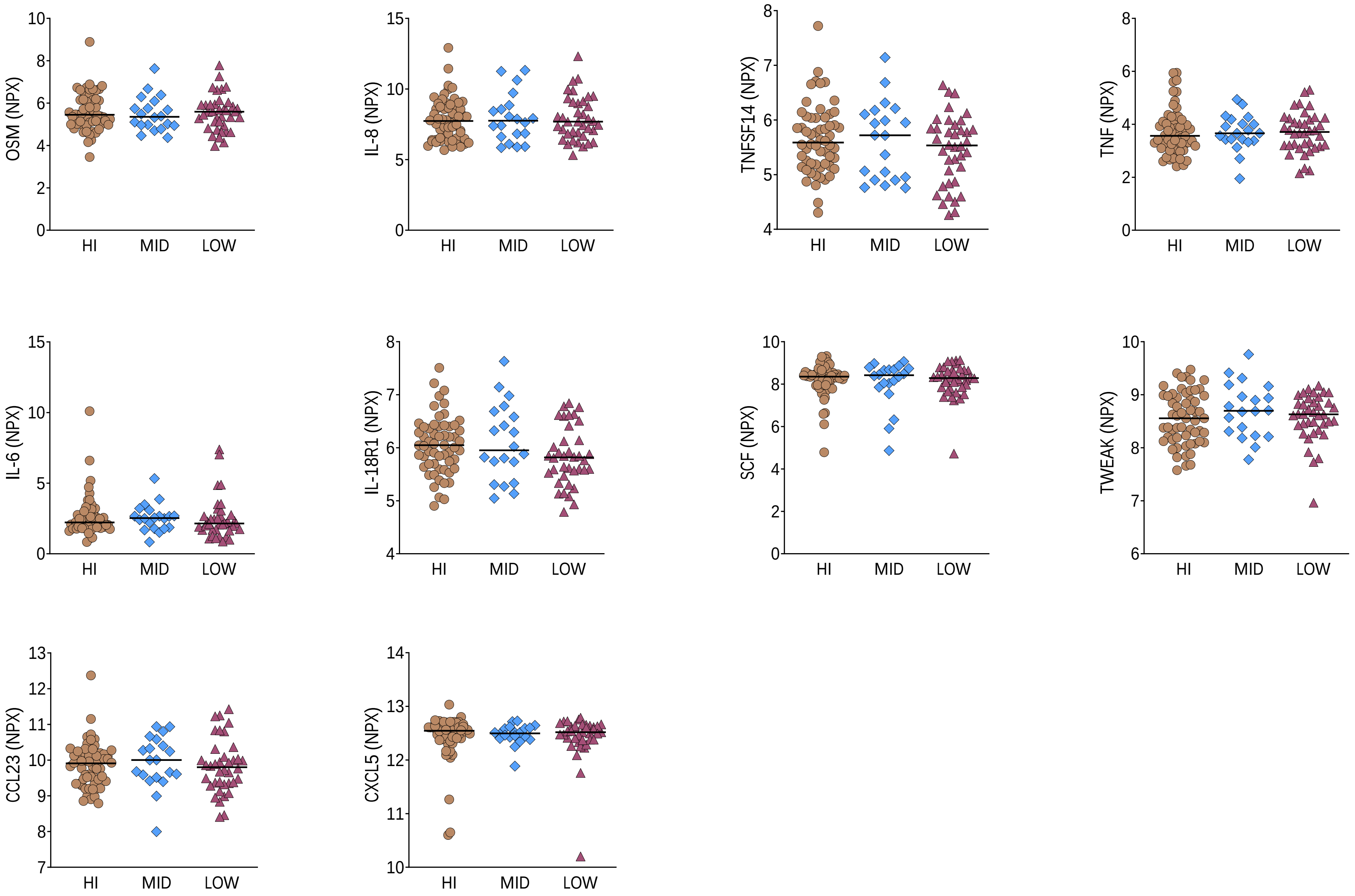

ESM Figure 5. *Inflammation-related proteins within the diabetes subgroup.* Analysis of ten differentially expressed inflammatory-related proteins (IRPs) across the diabetes subgroups of the omics subcohort was performed. Symbol plots show the protein expression levels and the y-axis represents normalized protein expression (NPX) values. The Kruskal-Wallis test was performed for group comparisons, and Dunn's correction was applied for multiple comparisons. Statistically significant differences between groups are indicated *, $p<0.05$; **, $p<0.01$; ***, $p<0.001$
